# Applicability of targeted metabolomics for the prediction of feed efficiency groups and metrics in dairy sheep

**DOI:** 10.3168/jdsc.2025-0905

**Published:** 2026-04-09

**Authors:** P.A.S. Fonseca, P. Frutos, L. Galucci, P.G. Toral, G. Hervás

**Affiliations:** Instituto de Ganadería de Montaña (CSIC-University of León), Finca Marzanas s/n, 24346 Grulleros, León, Spain

## Abstract

Identifying animals with high feed efficiency (FE) can significantly reduce production costs and environmental impact and improve profitability in livestock systems. Previous studies demonstrated that protein metabolism played a prominent role in predicting FE profiles in the Assaf breed. Consequently, a deeper investigation of the potential for protein-related metabolites to act as biomarkers for FE profile could help to determine a reduced subset of metabolites for FE prediction. In this study, 40 Assaf ewes were evaluated for FE using 2 metrics: residual feed intake (RFI) and feed efficiency index (FEI). Animals consistently classified at the extremes of both metrics were grouped as 6 lower (L-FE) and 6 higher (H-FE) FE individuals. Targeted plasma metabolomics, identified through ultra-performance liquid chromatography with multiple reaction monitoring tandem mass spectrometry with isotope-labeled target metabolites, was used to assess the potential of various metabolites related to protein metabolism to classify FE groups and predict FE metrics. Partial least squares discriminant analysis identified 9 metabolites with high variable importance in projection (>1.5) that separated L-FE and H-FE groups. In leave-one-out cross-validation, the model based on the first principal component (PC1) achieved Pearson correlations of 0.745 for RFI and 0.920 for FEI with observed values. Subsequently, the dimensionality of these data was reduced via principal component analysis, and PC1 was used in linear models to predict RFI and FEI. The functional profile of the selected metabolites reinforced the central role of protein metabolism, especially AA transport and utilization, in shaping FE phenotypes in dairy ewes. These findings enhance our understanding of protein metabolism signatures linked to FE and support the development of cost-effective tools (when compared with whole metabolomics) for selecting high-efficiency animals in ruminant production systems.

## Highlights

• Targeted protein-metabolite panel predicts FE in ewes.
• 12 variable importance in projection > 1.5 metabolites separate high- versus low-FE groups.
• Metabolites predict FE: r = 0.75 (RFI), r = 0.92 (FEI) in leave-one-out cross-validation (LOOCV).
• Amino acid transport and utilization are key pathways for FE.
• Reduced panels cut costs versus untargeted metabolomics for selection.

## Graphical Abstract

**Figure:**
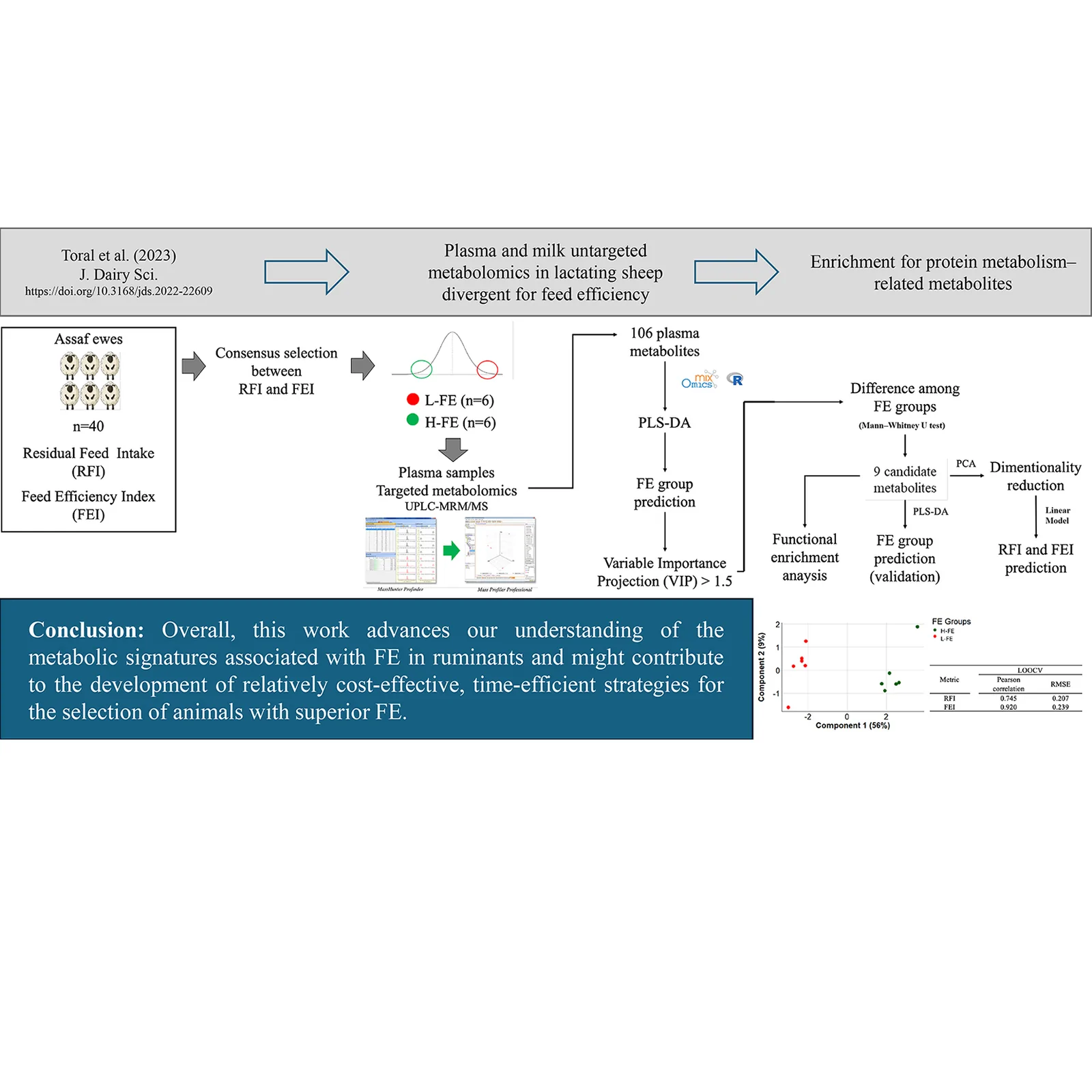
**Summary:** Enhancing feed efficiency (FE) in dairy sheep is a key strategy to reduce feed inputs, production costs, and environmental burden while maintaining animal productivity and farm profitability. Previous metabolomics studies in Assaf ewes highlighted protein metabolism as one of the main biological pathways associated with differences in FE. In this context, this study focused on a targeted panel of plasma metabolites involved in amino acid transport, utilization, and related protein-metabolism processes. Forty ewes were classified using residual feed intake (RFI) and FE index, and animals consistently ranked at the extremes were compared. The results showed that 9 protein-related metabolites were able to distinguish high- and low-efficiency ewes. These findings reinforce the importance of protein metabolism, particularly amino acid transport and utilization, in the biological regulation of FE. Overall, this work supports targeted metabolomics as a promising and practical approach to prospect biomarkers associated with FE in dairy sheep.

The growing global population has led to a rising demand for nutrient-rich food (FAO, 2018). In the livestock production chain, feed represents the largest costs (e.g., ∼66% in representative dairy sheep farms in Spain; RENGRATI, 2021). Feed efficiency (**FE**) describes the amount of feed required to produce a unit of output. Identifying and selecting animals with greater FE can reduce production costs, environmental impact, and improve profitability (Connor, 2015; Madilindi et al., 2022). However, estimating FE metrics, such as conversion ratios or residual-based traits, depends on individual feed intake records, which are costly and time consuming to obtain.

Recent studies have explored omics technologies for classifying FE groups (Martin et al., 2021; Toral et al., 2023; Nunes et al., 2024; Suárez-Vega et al., 2024) and predicting FE metrics in livestock species (Marina et al., 2024; Fonseca et al., 2025). Among these approaches, metabolomics is particularly useful for biomarker discovery because it captures the end products of cellular processes and provides a direct snapshot of physiological state (Griffiths et al., 2010; Peng et al., 2015). Previous studies from our group successfully applied untargeted milk and plasma metabolomics to predict FE groups and FE metrics in Assaf sheep (Toral et al., 2023; Marina et al., 2024), highlighting a prominent role of protein metabolism. Amino acid metabolism is essential for major production-related traits in ruminants, including growth and lactation (Kung and Rode, 1996). In addition, specific AA have been proposed as FE biomarkers because they reflect variation in protein synthesis, energy metabolism, muscle mass, protein degradation, ureagenesis, and nitrogen-use efficiency (Nunes et al., 2024).

Targeted metabolomics enables the evaluation of specific candidate metabolites and is therefore a useful biomarker discovery strategy (Griffiths et al., 2010). Here, we evaluated whether a reduced panel of plasma metabolites related to protein metabolism, previously highlighted in Assaf sheep (Toral et al., 2023), could discriminate divergent FE phenotypes and predict quantitative FE metrics. In this context, this study aimed to evaluate whether a reduced panel of plasma metabolites related to protein metabolism in Assaf ewes could both discriminate animals with divergent FE phenotypes and predict quantitative FE metrics.

Housing, performance, and FE calculations are fully described in Toral et al. (2021). Briefly, 40 Assaf ewes in early lactation were housed individually, fed ad libitum a 50:50 TMR, and milked twice daily. Individual DMI, milk yield, and BW were recorded for 3 weeks to calculate residual feed intake (**RFI**) and feed efficiency index (**FEI**).

These 2 metrics were used to classify the ewes in higher and lower-efficiency groups (**H-FE** and **L-FE**, respectively). As described previously (Toral et al., 2021), higher FE was associated with lower FEI and RFI values, averaging −0.29 for H-FE and 0.81 for L-FE ewes for FEI, and with an average of −0.16 and 0.18 for H-FE and L-FE, respectively, for RFI values. Both indexes showed a strong correlation (r = 0.69, *P* < 0.01). The H-FE and L-FE groups were significantly different for both RFI and FEI metrics based on a *t*-test (*P* < 0.001). Comparing the H-FE and L-FE groups across both metrics resulted in the identification of 6 ewes consistently classified as H-FE and 6 as L-FE by both methods. These 12 consensus ewes were used in the analyses performed to identify differentially abundant metabolites, cluster the FE groups, and predict the FE metrics.

On the last day of the trial, before the morning milking and administration of the feed, blood samples were collected into lithium heparin tubes (BD Vacutainer, Plymouth, UK) and centrifuged (1,811 × *g*) at 4°C for 10 min. Aliquots were placed on ice after sampling and further stored at −80°C until submitted for targeted metabolomics analysis, which was performed using 2 ultra-performance liquid chromatography with multiple reaction monitoring tandem mass spectrometry (**UPLC-MRM/MS**)-based protocols with isotope-labeled internal standards (**IS**; The Metabolomics Innovation Centre, TMIC, Victoria, BC, Canada). Internal standard solutions were prepared in either 90% or 80% acetonitrile, containing 35 or 5 labeled compounds for each one of the 2 UPLC-MRM/MS protocols performed. Stock solutions of target metabolites were diluted in water-IS mixtures (1:4 or 1:9, vol/vol) to create 10-point calibration curves ranging from 0.0001 to 100 nmol/mL. For sample preparation, 20 μL of plasma was mixed with 80 or 180 μL of IS solution for the first and second methods, vortexed, sonicated, and centrifuged (21,000 × *g* at 5°C). In one approach, a 50-μL supernatant aliquot was derivatized with dansyl chloride and borate buffer (pH 9), incubated at 40°C for 30 minutes, and analyzed using a Waters Acquity UPLC coupled to a Sciex QTRAP 6500 Plus mass spectrometer (Sciex, Framingham, MA). Chromatographic separation was performed on a C18 column (2.1 × 150 mm, 1.8 μm) with a binary solvent system—0.1% formic acid in water (A) and acetonitrile:isopropanol (1:1, B)—under a 21-min gradient (20% to 90% B) at 55°C and 0.35 mL/min. In the second approach, a 5-μL aliquot of the supernatant was injected directly into an Agilent 1290 UPLC system coupled to an Agilent 6495C triple quadrupole mass spectrometer (Agilent Technologies, Santa Clara, CA). Separation was achieved on a HILIC column (2.1 × 100 mm, 1.7 μm) using 0.1% formic acid in water (A) and acetonitrile (B), with a gradient from 90% to 20% B over 15 min at 40°C and 0.3 mL/min. For quantifying high-abundance AA, both calibration and sample solutions were diluted 10-fold with 50% acetonitrile and reanalyzed. Metabolite concentrations were calculated using IS calibration by interpolating analyte-to-IS peak area ratios against compound-specific linear regression curves.

Partial least squares discriminant analysis (**PLS-DA**) was conducted as an initial feature selection strategy using the R package mixOmics (Rohart et al., 2017) to assess the ability of selected features to distinguish between H-FE and L-FE groups. The area under the curve (**AUC**) was calculated to quantify the performance of the classification of the FE groups by PLS-DA. The performance of the PLS-DA model to classify the RFI and FEI groups was further evaluated using the mean error rate and the cross-validated squared correlation coefficient (**Q^2^**), based on the first 2 principal components (**PC**) and 5-fold cross-validation. Additionally, a permutation test, using 500 tests, was used to assess if the performance obtained in each model is better than could be obtained by chance. The significance of the permutation test was determined based on the *P*-value of the Q^2^ values obtained across the permutations. After the group assignment, variable importance in projection (**VIP**) scores were computed to identify influential predictors in the model. Features with VIP scores >1.5 were selected from the PLS-DA.

The differences between the 2 FE groups in the abundance of the subset of metabolites with a VIP >1.5 were evaluated using the Wilcoxon rank sum exact test in R. Significant differences were considered based on a 5% false discovery rate (**FDR**) threshold. In addition, Pearson correlation coefficients were calculated pairwise among the selected metabolites. To reduce pairwise correlations below a threshold of 0.8, highly correlated metabolites were removed using the findCorrelation function from the caret package (Kuhn, 2008). This final subset of metabolites was used to fit a new PLS-DA model and evaluate the improvement in classification (based on the percentage of variance explained by the components). The same final subset of selected metabolites was used in a principal component analysis (**PCA**) to reduce the dimensionality of the metabolites dataset and fit the PC scores in linear models for the predictions of RFI and FEI values. The number of PC to include in the model was determined using a forward selection approach, where components were added iteratively, from the first onward, until the cumulative explained variance reached at least 80%. The final model was defined based on the higher adjusted R^2^. The predictive performance of these models was evaluated using Pearson correlation coefficients and root mean squared errors (**RMSE**) between the predicted and the actual values. The model generalization was also assessed using a leave-one-out cross-validation (**LOOCV**) based on the mean Pearson correlation coefficients and RMSE.

Functional enrichment analysis, based on metabolic pathways, was conducted for the subset of selected metabolites, with a VIP >1.5, using MetaboAnalyst 6.0 (Pang et al., 2024). The Relational database of Metabolomic Pathways database (RaMP-DB; https://rampdb.nih.gov/) was used to access the association between the selected metabolites and Kyoto Encyclopedia of Genes and Genomes (KEGG), Reactome, and WikiPathways metabolic pathways. An over-representation analysis (**ORA**) was performed for those pathways with at least 2 associated metabolites and enriched pathways were determined based on a 5% FDR threshold. The reference background during the ORA was set as the metabolites included in the targeted metabolomics of serum samples.

Initially, 153 metabolites (Supplemental Table S1, see Notes) were processed and quantified in plasma, from which 50 were filtered out due to low quantifications across all samples (quantification mean below 0.1 nmol/mL). Therefore, a dataset composed of 103 metabolites was included in the PLS-DA (Supplemental Table S1). The mean and the SD (in nmol/mL) and the variation coefficient obtained for each metabolite in the H-FE and L-FE groups are shown in Supplemental Table S2 (see Notes).

The mean (± SD) for the H-FE and L-FE evaluated in this study were 0.263 kg (± 0.201) and −0.264 kg (± 0.132) for RFI and 0.201 kg (± 0.258) and 0.132 kg (± 0.153) for FEI. The PLS-DA results obtained using the 103 metabolites indicated a perfect separation of the FE groups, with an AUC of 1 and the first 2 components explaining 19% and 14% of the total variance, respectively (Figure 1). The cross-validation of this model resulted in a mean Q^2^ of 0.875 (*P*-value permutation test equal to 0.008), a mean error rate of 0.125, and a cumulative sum of squares of the Y variables explained by the latent variables (**R^2^Y**) of 0.666. A total of 12 metabolites presented a VIP >1.5: ℽ-aminobutyric acid (**GABA**), diaminopimelic acid, 3-hydroxy-l-tyrosine (**L-DOPA**), glycine, isoleucine, leucine, methionine, ornithine, phenylalanine, proline, purine, and uric acid. The pairwise Pearson correlation coefficient analysis suggested the exclusion of leucine, phenylalanine, and uric acid to reduce the collinearity in the dataset. Therefore, 9 metabolites were retained for the second round of PLS-DA. This model also resulted in an AUC = 1. However, the first component of this model explained 56% of the total variance, whereas the second component explained 9% of the total variance (Figure 1). The cross-validated coefficient of determination of this model was Q^2^ = 1 (*P*-value permutation test <0.001), a mean error rate of 0, and a R^2^Y of 1. The Wilcoxon rank sum exact test indicated significant differences (*P* < 0.05, FDR <0.05) between the H-FE and L-FE groups for the 9 selected metabolites. The direction of the differences between the FE groups for the 9 selected metabolites and the statistical significance are shown in Table 1. Interestingly, all 9 metabolites showed smaller means for the abundances in the L-FE group. The direction of the differences between the FE groups for the 103 metabolites dataset can be assessed in Supplemental Table S2. The PCA indicated that 3 PC were enough to reach at least 80% of the cumulative explained variance, with the first, second, and third PC explaining approximately 56%, 12%, and 11%, respectively (Supplemental Figure S1, see Notes). Among the models evaluated using 1, 2, 3, and 4 PC, the model including the first PC showed the highest adjusted R^2^ for both RFI (0.65) and FEI (0.88). Therefore, a simple linear model composed of the first PC from the PCA performed using the 9 selected metabolites was fitted to predict RFI and FEI values. The RFI model presented an intercept of −0.00075 (*P* = 0.99), the PC1 coefficient of 0.102 (*P* < 0.001), and a residual standard error equal to 0.189. The FEI model presented an intercept of 0.274 (*P* = 0.001), a PC1 coefficient of 0.232 (*P* < 0.001), and a residual standard error equal to 0.216. The analysis of the residual distribution for both models did not show deviations from normality based on the Shapiro–Wilk test (*P* > 0.05). In general, good predictive performance was obtained in both models, with the predictions for FEI resulting in higher Pearson correlation coefficients (Table 2). The LOOCV results indicated good generalization potential for the models, as the Pearson correlation coefficients and RMSE values were similar to those obtained using the full dataset, whereas the Q^2^ indicated better generalization for the FEI values (Table 2).

**Figure 1:**
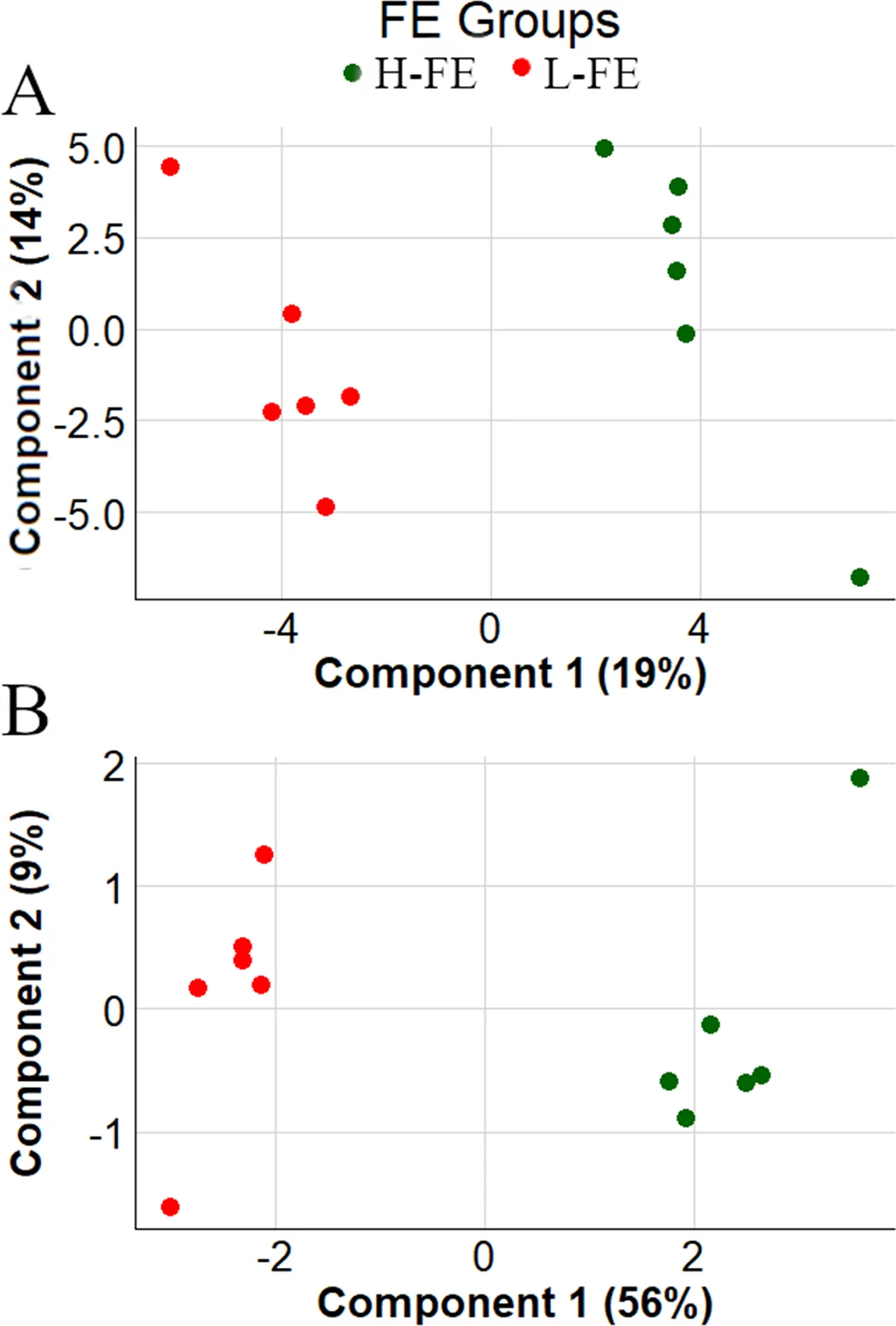
Clustering of feed efficiency (FE) groups obtained via partial least squares discriminant analysis (PLS-DA) for the complete dataset of 153 metabolites (A) and the subset of 9 metabolites with variable importance in projection (VIP) scores higher than 1.5 obtained in the first PLS-DA (B). The green dots represent the ewes with higher FE (H-FE), and the red dots represent the ewes with lower FE (L-FE).

**Table 1:** Mean (± SD) in nmol/mL, CV, raw *P*-values, and false discovery rate (FDR) adjusted *P*-values for the 9 selected metabolites in the plasma of Assaf ewes contrasting for divergent (high [H] and low [L]) feed efficiency (FE) groups

Metabolite	Mean (± SD)		CV		*P*-value	FDR
	H-FE	L-FE	H-FE	L-FE		
Aminobutyric acid-α	8.80 (± 3.85)	15.17 (± 4.99)	43.71	32.91	0.026	0.038
Diaminopimelic acid	0.22 (± 0.05)	0.50 (± 0.27)	24.13	54.17	0.037	0.041
L-DOPA methyl ester	0.85 (± 0.09)	0.98 (± 0.08)	10.27	8.58	0.037	0.041
Glycine	303.75 (± 56.58)	374.42 (± 32.73)	18.63	8.74	0.015	0.034
Isoleucine	102.42 (± 15.89)	146.4 (± 12.58)	15.51	8.59	0.002	0.009
Methionine	12.17 (± 2.82)	16.75 (± 2.89)	23.14	17.27	0.041	0.041
Ornithine	46.46 (± 26.37)	95.63 (± 43.09)	56.76	45.06	0.026	0.039
Proline	197.90 (± 28.44)	293.83 (± 20.10)	14.37	6.84	0.002	0.010
Purine	1.46 (± 0.26)	1.84 (± 0.17)	17.78	9.48	0.015	0.034

**Table 2:** Predictive performance of the first principal component obtained in the principal component analysis for the 9 selected metabolites in the plasma of Assaf ewes for residual feed intake (RFI) and feed efficiency index (FEI)^1^

Metric	Model performance		LOOCV		
	Pearson correlation	RMSE	Pearson correlation	RMSE	Q^2^
RFI	0.825	0.173	0.745	0.207	0.513
FEI	0.946	0.197	0.920	0.239	0.763

1 LOOCV = leave-one-out cross-validation; RMSE = root mean squared error; Q^2^ = cross-validated coefficient of determination.

The ORA results indicated that no pathways were significantly enriched at a 5% FDR threshold (Supplemental Table S3, see Notes). However, analysis at the raw *P*-value level revealed an interesting pattern of biological processes associated with the selected metabolites. For instance, the top 15 pathways ranked by raw *P*-values suggested a close connection between these metabolites and AA transport, particularly SLC-mediated transmembrane transport (Supplemental Table S3).

The predictive models, PLS-DA and linear model, exhibited good predictive performance for the FE groups using the 9 selected metabolites. In the case of PLS-DA, the LOOCV analysis indicated a good generalization of the model, which perfectly separates the FE groups. The linear model used to predict the RFI and FEI values, based on PC1 from the PCA of the 9 metabolites, resulted in a good predictive performance. The LOOCV suggested a higher Pearson correlation between the actual and predicted values for FEI in comparison to RFI. It is important to emphasize that the results reported here were generated from a limited sample size, and validation in a larger and more heterogeneous population is required before broader generalization can be supported. This requirement is further underscored by the Q^2^ and AUC values observed for the reduced metabolite subset in the PLS-DA, which may indicate possible overfitting and should therefore be interpreted with caution and investigated further.

Before presenting the biological interpretation of the selected metabolites, it should be noted that all 9 metabolites retained in the final reduced panel exhibited lower mean abundance in the L-FE group than in the H-FE group. Accordingly, the following discussion focuses on metabolites that were, on average, more abundant in the more feed-efficient animals. In addition, because these results were obtained from association-based analyses, they should not be interpreted as evidence of causality. Thus, we intentionally avoided stronger mechanistic speculation and the formulation of definitive biological pathways underlying FE divergence. Instead, the following discussion aims to provide a cautious and structured interpretation of the identified targeted metabolite signature in light of previous studies and the statistical patterns observed in the present dataset. The evidence of the relevance of protein metabolism, with emphasis on the AA transport, to the FE classification was previously described in Assaf ewes based on the investigation through other omics technologies, such as genomics, epigenomics, and transcriptomics (Toral et al., 2023; Marina et al., 2024; Suárez-Vega et al., 2024; Fonseca et al., 2025). In the current study, the metabolic pathway enrichment analysis (performed using the 9 selected metabolites shown in Table 1) reinforced the relationship between the selected metabolites and AA transport, mainly across the membrane and SLC-mediated transport. Previous studies highlighted a prominent role of protein turnover and nutrient transporters across the ruminal epithelium in the FE control (Cantalapiedra-Hijar et al., 2018; Elolimy et al., 2019; Nunes et al., 2024). Enhanced understanding and targeting of AA transport and signaling pathways can optimize protein anabolism (Poncet and Taylor, 2013), consequently offering new strategies to improve FE in ruminants.

Among the 9 selected metabolites, 5 were AA: glycine, isoleucine, methionine, ornithine, and proline. Gluconeogenic AA such as glycine and proline may influence differences in energy availability between animals divergent for FE (Karisa et al., 2014). Higher plasma ornithine concentration in more FE growing heifers has been hypothesized to reflect a slower urea cycle (Jorge-Smeding et al., 2019). In ruminants, especially cattle, rumen-protected methionine supplementation has been associated with increased milk yield and milk components, slight reductions in DMI, and improved nitrogen utilization (Patton, 2010; Chen et al., 2011; Giallongo et al., 2016). In contrast, inefficient ruminants showed greater nitrogen metabolism activity linked to methionine-related pathways in the rumen microbiome (Kruger Ben Shabat et al., 2016; Li and Guan, 2017).

The GABA supplementation in dairy cows has been associated with greater milk yield, milk solids, and DMI; reduced nitrogen emissions; and improved intake, although not necessarily with direct FE improvements under heat stress (Wang et al., 2013; Cheng et al., 2014). Diaminopimelic acid is considered a rumen bacterial marker and a proxy for rumen protein synthesis (Dufva et al., 1982; Illg and Stern, 1994). 3-Hydroxy-l-tyrosine is a precursor of dopamine (Dayan and Finberg, 2003; González-Sepúlveda et al., 2022), and the gut-brain dopamine axis has been suggested to contribute to intake regulation, energy balance, and glucose metabolism (Ren et al., 2010; de Araujo et al., 2012; Mietlicki-Baase et al., 2015).

By integrating metabolomic data with predictive modeling approaches, we demonstrated that both PLS-DA and linear models effectively classify animals into divergent FE groups and predict FE metrics with high accuracy and low prediction error. A subset of 9 metabolites seems to work as robust biomarkers for FE in Assaf ewes. These results reinforce the pivotal role of protein metabolism, particularly AA transport and utilization. Overall, this work supports the value of a reduced targeted metabolite panel associated with FE in ruminants and underscores its potential application as a relatively cost-effective and time-efficient strategy for FE characterization and for identifying animals with superior FE, rather than as a general exploratory metabolomics approach.

## References

[bib1] Cantalapiedra-Hijar G., Abo-Ismail M., Carstens G.E., Guan L.L., Hegarty R., Kenny D.A., Mcgee M., Plastow G., Relling A., Ortigues-Marty I. (2018). Review: Biological determinants of between-animal variation in feed efficiency of growing beef cattle. Animal.

[bib2] Chen Z.H., Broderick G.A., Luchini N.D., Sloan B.K., Devillard E. (2011). Effect of feeding different sources of rumen-protected methionine on milk production and N-utilization in lactating dairy cows. J. Dairy Sci..

[bib3] Cheng J.B., Bu D.P., Wang J.Q., Sun X.Z., Pan L., Zhou L.Y., Liu W. (2014). Effects of rumen-protected γ-aminobutyric acid on performance and nutrient digestibility in heat-stressed dairy cows. J. Dairy Sci..

[bib4] Connor E.E. (2015). Invited review: Improving feed efficiency in dairy production: Challenges and possibilities. Animal.

[bib5] Dayan L., Finberg J.P.M. (2003). L-DOPA increases noradrenaline turnover in central and peripheral nervous systems. Neuropharmacology.

[bib6] de Araujo I.E., Ferreira J.G., Tellez L.A., Ren X., Yeckel C.W. (2012). The gut-brain dopamine axis: A regulatory system for caloric intake. Physiol. Behav..

[bib7] Dufva G.S., Bartley E.E., Arambel M.J., Nagaraja T.G., Dennis S.M., Galitzer S.J., Dayton A.D. (1982). Diaminopimelic acid content of feeds and rumen bacteria and its usefulness as a rumen bacterial marker. J. Dairy Sci..

[bib8] Elolimy A.A., Abdel-Hamied E., Hu L., McCann J.C., Shike D.W., Loor J.J. (2019). Rapid Communication: Residual feed intake in beef cattle is associated with differences in protein turnover and nutrient transporters in ruminal epithelium. J. Anim. Sci..

[bib9] FAO (Food and Agriculture Organization of the United Nations). 2018. The future of food and agriculture: Alternative pathways to 2050. Food and Agriculture Organization of the United Nations, Rome, Italy.

[bib10] Fonseca P.A.de S., Suarez-Vega A., Esteban-Blanco C., Marina H., Pelayo R., Gutiérrez-Gil B., Arranz J.-J. (2025). Integration of epigenomic and genomic data to predict residual feed intake and the feed conversion ratio in dairy sheep via machine learning algorithms. BMC Genomics.

[bib11] Giallongo F., Harper M.T., Oh J., Lopes J.C., Lapierre H., Patton R.A., Parys C., Shinzato I., Hristov A.N. (2016). Effects of rumen-protected methionine, lysine, and histidine on lactation performance of dairy cows. J. Dairy Sci..

[bib12] González-Sepúlveda M., Omar M.Y., Hamdon S., Ma G., Rosell-Vilar S., Raivio N., Abass D., Martínez-Rivas A., Vila M., Giraldo J., Carrascal M., Abián J., Gil C., Sabriá J., Ortiz J., Moreno-Delgado D. (2022). Spontaneous changes in brain striatal dopamine synthesis and storage dynamics ex vivo reveal end-product feedback-inhibition of tyrosine hydroxylase. Neuropharmacology.

[bib13] Griffiths W.J., Koal T., Wang Y., Kohl M., Enot D.P., Deigner H.P. (2010). Targeted metabolomics for biomarker discovery. Angew. Chem. Int. Ed. Engl..

[bib14] Illg D.J., Stern M.D. (1994). In vitro and in vivo comparisons of diaminopimelic acid and purines for estimating protein synthesis in the rumen. Anim. Feed Sci. Technol..

[bib15] Jorge-Smeding E., Renand G., Centeno D., Pétéra M., Durand S., Polakof S., Cantalapiedra-Hijar G. (2019). Metabolomics reveals changes in urea cycle associated to residual feed intake in growing heifers. EAAP Scientific Series.

[bib16] Karisa B.K., Thomson J., Wang Z., Li C., Montanholi Y.R., Miller S.P., Moore S.S., Plastow G.S. (2014). Plasma metabolites associated with residual feed intake and other productivity performance traits in beef cattle. Livest. Sci..

[bib17] Kruger Ben Shabat S., Sasson G., Doron-Faigenboim A., Durman T., Yaacoby S., Berg Miller M.E., White B.A., Shterzer N., Mizrahi I. (2016). Specific microbiome-dependent mechanisms underlie the energy harvest efficiency of ruminants. ISME J..

[bib18] Kuhn M. (2008). Building predictive models in R using the caret package. J. Stat. Softw..

[bib19] Kung L., Rode L.M. (1996). Amino acid metabolism in ruminants. Anim. Feed Sci. Technol..

[bib20] Li F., Guan L.L. (2017). Metatranscriptomic profiling reveals linkages between the active rumen microbiome and feed efficiency in beef cattle. Appl. Environ. Microbiol..

[bib21] Madilindi M.A., Zishiri O.T., Dube B., Banga C.B. (2022). Technological advances in genetic improvement of feed efficiency in dairy cattle: A review. Livest. Sci..

[bib22] Marina H., Arranz J.J., Suárez-Vega A., Pelayo R., Gutiérrez-Gil B., Toral P.G., Hervás G., Frutos P., Fonseca P.A.S. (2024). Assessment of milk metabolites as biomarkers for predicting feed efficiency in dairy sheep. J. Dairy Sci..

[bib23] Martin M.J., Dórea J.R.R., Borchers M.R., Wallace R.L., Bertics S.J., DeNise S.K., Weigel K.A., White H.M. (2021). Comparison of methods to predict feed intake and residual feed intake using behavioral and metabolite data in addition to classical performance variables. J. Dairy Sci..

[bib24] Mietlicki-Baase E.G., Reiner D.J., Cone J.J., Olivos D.R., McGrath L.E., Zimmer D.J., Roitman M.F., Hayes M.R. (2015). Amylin modulates the mesolimbic dopamine system to control energy balance. Neuropsychopharmacology.

[bib25] Nunes A.T., Faleiros C.A., Poleti M.D., Novais F.J., López-Hernández Y., Mandal R., Wishart D.S., Fukumasu H. (2024). Unraveling ruminant feed efficiency through metabolomics: A systematic review. Metabolites.

[bib26] Pang Z., Lu Y., Zhou G., Hui F., Xu L., Viau C., Spigelman A.F., MacDonald P.E., Wishart D.S., Li S., Xia J. (2024). MetaboAnalyst 6.0: Towards a unified platform for metabolomics data processing, analysis and interpretation. Nucleic Acids Res..

[bib27] Patton R.A. (2010). Effect of rumen-protected methionine on feed intake, milk production, true milk protein concentration, and true milk protein yield, and the factors that influence these effects: A meta-analysis. J. Dairy Sci..

[bib28] Peng B., Li H., Peng X.X. (2015). Functional metabolomics: From biomarker discovery to metabolome reprogramming. Protein Cell.

[bib29] Poncet N., Taylor P.M. (2013). The role of amino acid transporters in nutrition. Curr. Opin. Clin. Nutr. Metab. Care.

[bib30] Ren X., Ferreira J.G., Zhou L., Shammah-Lagnado S.J., Yeckel C.W., de Araujo I.E. (2010). Nutrient selection in the absence of taste receptor signaling. J. Neurosci..

[bib31] RENGRATI. 2021. Red Nacional de Granjas Típicas. Informe de Resultados Económicos de Ovino de Leche.

[bib32] Rohart F., Gautier B., Singh A., Lê Cao K.A. (2017). mixOmics: An R package for 'omics feature selection and multiple data integration. PLOS Comput. Biol..

[bib33] Suárez-Vega A., Gutiérrez-Gil B., Fonseca P.A.S., Hervás G., Pelayo R., Toral P.G., Marina H., de Frutos P., Arranz J.J. (2024). Milk transcriptome biomarker identification to enhance feed efficiency and reduce nutritional costs in dairy ewes. Animal.

[bib34] Toral P.G., Abecia L., Hervás G., Yáñez-Ruiz D.R., Frutos P. (2023). Plasma and milk metabolomics in lactating sheep divergent for feed efficiency. J. Dairy Sci..

[bib35] Toral P.G., Hervás G., Fernández-Díez C., Belenguer A., Frutos P. (2021). Rumen biohydrogenation and milk fatty acid profile in dairy ewes divergent for feed efficiency. J. Dairy Sci..

[bib36] Wang D.M., Wang C., Liu H.Y., Liu J.X., Ferguson J.D. (2013). Effects of rumen-protected γ-aminobutyric acid on feed intake, lactation performance, and antioxidative status in early lactating dairy cows. J. Dairy Sci..

